# Ethical gaps in closed-loop neurotechnology: a scoping review

**DOI:** 10.1038/s41746-025-01908-4

**Published:** 2025-08-08

**Authors:** Lea Haag, Georg Starke, Markus Ploner, Marcello Ienca

**Affiliations:** 1 Laboratory of Ethics of AI & Neuroscience, Institute of History and Ethics in Medicine, School of Medicine and Health, Technical University of Munich (TUM), Munich, Germany; 2 Center for Interdisciplinary Pain Medicine and Department of Neurology, School of Medicine and Health, Technical University of Munich (TUM), Munich, Germany

**Keywords:** Policy, Ethics, Neuroscience, Neurology

## Abstract

Closed-loop (CL) neurotechnology, which dynamically adapts to patients’ neural states, offers new opportunities for treating neurological and psychiatric disorders. However, its real-time feedback mechanisms raise critical ethical challenges. This scoping review assesses whether and how clinical studies involving CL neurotechnologies address ethical concerns. We analyzed peer-reviewed research on human participants to evaluate both the presence and depth of ethical engagement. Despite the prominence of CL systems in neuroethical discourse, explicit ethical assessments remain rare. Ethical issues are typically addressed only implicitly, folded into technical or procedural discussions without structured analysis. Most notably, our findings reveal a persistent gap between regulatory compliance and meaningful ethical reflection. To address this, we propose empirically grounded, community-responsive recommendations to strengthen ethical oversight in this field. These recommendations aim to support governance frameworks that are context-sensitive, reflexive, and capable of addressing the complex ethical terrain introduced by adaptive neurotechnologies.

## Introduction

Closed-loop (CL) systems have emerged as a pioneering medical technology across various areas of healthcare such as endocrinology —where CL insulin delivery systems are transforming diabetes management^1,2^ — and, most recently, neurology^3^ and psychiatry^4^. Also referred to as *adaptive*^5^ or *responsive*^6^ technologies, these systems operate by continuously monitoring physiological inputs, processing the data through advanced algorithms, and dynamically adjusting outputs in real time to achieve desired outcomes. This approach enables not only precise control and enhanced efficacy but also personalized treatment tailored to each patient’s momentary physiological state^7^.

Within neurology and psychiatry, CL systems offer promising new treatment modalities. In clinical neurology, these systems facilitate real-time monitoring and intervention. A notable example is the FDA-approved responsive neurostimulation (RNS) system, which utilizes intracranial electroencephalography (iEEG) to detect epileptiform activity and deliver targeted stimulation to prevent seizures^8^. Another key application is adaptive deep brain stimulation (aDBS), which continuously tracks neural fluctuations—such as beta-band oscillations in local field potentials (LFPs)—and dynamically modulates stimulation parameters at patient-specific targets like the subthalamic nucleus. This approach has demonstrated significant improvements in symptom management for Parkinson’s disease^9,10^. These precision medicine interventions optimize therapeutic outcomes while minimizing risks and adverse effects, offering substantial improvements in the treatment of neurological conditions such as Parkinson’s disease, epilepsy, and essential tremor.

In psychiatry, CL neurostimulation and neuromodulation systems are emerging as promising tools for treating complex conditions such as depression, schizophrenia, and obsessive-compulsive disorder. These systems leverage real-time neurophysiological monitoring techniques, such as electroencephalography (EEG), to detect abnormal brain activity associated with these disorders. Research has investigated various biomarkers, including specific EEG patterns^11^ and local field potentials (LFPs) recorded from brain regions such as the stria terminalis, cingulate cortex^12^, and subcallosal cingulate^13^, to assess symptom fluctuations and guide therapeutic interventions^14^. While EEG remains the predominant modality, recent studies have begun exploring LFPs as an alternative for capturing more nuanced neural activity underlying psychiatric conditions^12^. By continuously monitoring these biomarkers, CL systems can dynamically adjust electrical stimulation parameters, offering a more personalized and adaptive approach to treatment. This real-time feedback-driven modulation holds significant promise for improving psychiatric care outcomes^15,16^ and extending its applicability to related fields, such as chronic pain management^17^.

Despite the transformative potential of CL neurotechnologies, their integration into clinical practice raises several ethical concerns. While these systems enhance treatment precision and minimize side effects, their adaptive nature and reliance on artificial intelligence (AI) introduce novel challenges that require careful scrutiny^18–21^. For instance, the continuous real-time recording and processing of neural data further raises challenges for privacy and the patients’ right to be informed about when and how their data are collected and processed^19–23^. From an ethical standpoint, resolving these concerns requires more than regulatory compliance; it demands transparent communication, informed consent procedures tailored to adaptive systems, and principled deliberation on how to balance privacy protection with device functionality through frameworks such as proportionality and least-infringement. Furthermore, the integration of AI in CL neurotechnologies raises concerns about their potential impact on patients’ sense of self and identity, as these systems can autonomously modulate neural activity in ways that may blur the distinction between voluntary and externally driven actions^18,22^. The extent to which patients perceive these interventions as an extension of their own agency or as an external influence remains largely unexplored, warranting further investigation^24^. Finally, issues related to equitable access to CL interventions add another layer of complexity to the ethical landscape, as these applications are often resource-intensive and require specialized expertise, potentially exacerbating existing healthcare disparities and leaving underserved communities without access to these advanced therapeutic options^20^. While these ethical considerations are widely discussed in the neuroethical literature^19,21,25^, it remains unclear whether and to what extent they are addressed in actual clinical research practice.

To address this gap, this review investigates whether and how ethical issues are addressed in current clinical studies involving CL neurotechnologies. In addition to monitoring the prevalence of ethics-related language, we assess the depth, explicitness, and critical quality of ethical engagement in clinical reporting. Our goal is to determine not only what ethical dimensions are acknowledged but how they are integrated, justified, and acted upon. In doing so, our analysis also interrogates the relationship between regulatory compliance and deeper ethical reflection, identifying a recurring disconnect that limits the ethical robustness of current practices. To our knowledge, this is the first systematic synthesis of ethical engagement in clinical research on CL systems in human subjects. The review provides both a quantitative and qualitative account of ethical attention in the literature and aims to bridge the gap between theoretical debate and empirical research. Based on these findings, we present a set of empirically grounded, non-prescriptive recommendations to guide the responsible development and governance of CL neurotechnologies. These recommendations are not abstract or predetermined but emerge from the ethical blind spots and patterns identified in the literature. Ultimately, the review contributes to a more reflective, inclusive, and practically actionable ethical discourse in the rapidly evolving field of closed-loop brain technologies.

## Results

Among the 66 reviewed studies, one included a dedicated assessment of ethical considerations, suggesting that ethics is not currently a central focus in most ongoing clinical trials. Where ethical language did appear, it was primarily restricted to formal references to procedural compliance such as affirmations of institutional review board (IRB) approval or adherence to regulatory guidelines—rather than substantive ethical engagement. This suggests that ethics, when present, is framed procedurally rather than reflectively. Although explicit ethical discussions were rare, some studies implicitly addressed ethically significant issues such as patient autonomy, data privacy, and risk-benefit considerations, though these were typically discussed in technical or clinical terms, without being identified or developed as ethical concerns. In the following section, we present the five key ethical themes that emerged from our thematic coding. Table 1 provides an overview of the reviewed studies, categorized by system type, diagnosis, participant gender distribution, number of participants, and mean age at implantation.

**Table 1:** Overview of reviewed studies

System used	Diagnosis	Gender f:m	Number of participants	Mean age in years at implantation	Reference
DBS	Parkinsons´ disease	n.a.	8	54,00	^77^
DBS	Parkinsons´ disease	0:2	2	63,00	^28^
DBS	Parkinsons´ disease	0:1	1	51,00	^124^
DBS	Parkinsons´ disease	n.a.	8	59,10	^10^
DBS	Parkinsons´ disease	6:7	13	62,15	^73^
DBS	Parkinsons´ disease	0:4	4	53,30	^83^
DBS	Parkinsons´ disease; dystonia	5:6 PD; 3:4 Dystonia	18	61,80 PD; 58,14 Dystonia	^29^
DBS	Essential tremor	0:1	1	59,00	^30^
DBS	Parkinsons´ disease	2:3	5	59,20	^31^
DBS	Parkinsons´ disease	2:11	13	61,90	^78^
DBS	Essential tremor	0:2	2	77,00	^74^
DBS	Parkinsons´ disease	n.a.	13	56,80	^93^
DBS	Parkinsons´ disease	n.a.	3	n.a.	^82^
DBS	Tremor	0:4	4	65,50	^32^
DBS	Essential tremor	3:6	9	72,60	^79^
DBS	Essential tremor	5:5	10	72,70	^27^
DBS	Essential tremor	n.a.	3	76,30	^33^
DBS	Essential tremor	3:5	8	68,40	^34^
DBS	Essential tremor	0:3	3	73,70	^9^
DBS	Essential tremor	n.a.	3	n.a.	^75^
RNS	Epilepsy	2:12	14	38,20	^84^
RNS	Epilepsy	n.a.	7	33,40	^35^
RNS	Epilepsy	n.a.	3	45,70	^36^
RNS	Epilepsy	53:58	111	37,30	^37^
RNS	Epilepsy	63:63	126	30,40	^38^
RNS	Epilepsy	32:18	50	39,50	^39^
RNS	Epilepsy	0:1	1	11,00	^88^
RNS	Epilepsy	125:131	256	34,00	^8^
RNS	Epilepsy	125:131	256	34,00	^40^
RNS	Epilepsy	125:131	256	34,00	^41^
RNS	Epilepsy	92:99	191	34,90	^42^
RNS	Epilepsy	26:30	56	14,90	^81^
RNS	Epilepsy	3:0	3	37,30	^43^
RNS	Epilepsy	3:7	10	44,60	^44^
RNS	Epilepsy	3:3	6	n.a.	^64^
RNS	Epilepsy	91:100	191	34,90	^45^
RNS	Epilepsy	84:91	175	34,60	^46^
RNS	Epilepsy	n.a.	18	n.a.	^65^
VNS	Epilepsy	6:11	17	28,80	^47^
VNS	Epilepsy	9:5	14	36,30	^96^
VNS	Epilepsy	19:9	28	40,00	^48^
VNS	Epilepsy	0:1	1	29,00	^68^
VNS	Essential tremor	0:1	1	58,00	^49^
VNS	Epilepsy	19:11	30	34,70	^50^
VNS	Epilepsy	n.a.	46	14,00	^51^
VNS	Epilepsy	Open 31:39 + Closed 13:19	Open 70 + Closed 31	Open 34 + Closed 20	^85^
VNS	Epilepsy	5:10	15	31,00	^52^
VNS	Epilepsy	2:1	3	25,30	^53^
VNS	Epilepsy	19:11	30	34,70	^54^
VNS	Epilepsy	10:20	30	15,03	^55^
VNS	Epilepsy	7:13	20	11,70	^80^
VNS	Epilepsy	3:4 AspireSR; 4:3 Demipulse	7 AspireSR; 7 Demipulse	38 Aspire SR; 28 Demipulse	^56^
VNS	Epilepsy	10:5	15	34,20	^57^
VNS	Epilepsy	12:10	22	35,60	^58^
VNS	Epilepsy	9:8	17	40,20	^59^
VNS	Epilepsy	220:216	436	29,00	^60^
VNS	Epilepsy	15:11	26	37,10	^66^
VNS	Epilepsy	n.a.	5	n.a.	^61^
VNS	Epilepsy	19:12	31	39,60	^67^
fMRI, neurofeedback	Depression and healthy	Depression 8:7 healthy 6:6	15 depression; 12 healthy	27,3 depression and 25,4 healthy	^69^
EEG-based BCI system visual feedback	Healthy	0:20	20	22,10	^76^
CL Neuroprosthesis, neuromuscular stimulation	Stroke	6:12	18	56,00	^62^
CL Limb prosthesis electrical and vibrotactile sensory feedback	Bilateral transradial amputation	0:1	1	44,00	^94^
CL neuromuscular electrical stimulation	Healthy	n.a.	5	n.a.	^70^
Motion and EMG monitoring, audio-visual feeedback in VR	Healthy	n.a.	15	19-35	^71^
tDCS, proprioceptive feedback	Healthy	16:16	32	25,90	^72^

### Beneficence

Beneficence refers to the ethical principle of promoting well-being by maximizing benefits and minimizing risks and costs^26^. Among the many possible ways to understand this principle in the context of closed-loop neurotechnology, beneficence was primarily discussed in two ways in the reviewed literature: as a rationale for pursuing new treatment avenues, particularly when conventional therapies had failed, and as a justification for maintaining hope in otherwise limited clinical situations. Many studies emphasized that traditional treatments, whether pharmacological or stimulation-based, often loose efficacy over time or cause intolerable side effects, compromising patient well-being^27^. Consequently, CL systems were framed as a means to enhance therapeutic outcomes and uphold beneficence. Among the 66 reviewed studies, 38 explicitly cited the ineffectiveness of alternative treatments as a key motivation for investigating CL neurotechnologies^8,9,27–62^. Effectiveness limitations included non-tolerable side effects, diminishing efficacy, and complete treatment resistance. These barriers reinforced the role of CL neurotechnologies in optimizing therapeutic benefits through real-time adaptability and precision. In evaluating beneficence, two primary approaches emerged: therapeutic and preventative. Most studies focused on therapeutic interventions, aiming to treat existing conditions or enhance patient outcomes, whereas fewer explored preventative strategies to mitigate future risks. Notably, 11 studies involved children, and 62 examined last-resort interventions for vulnerable populations. While all reviewed studies examined the effectiveness of CL systems, only a minority assessed their impact on QoL. While all reviewed studies examined the effectiveness of CL systems, the ethical framing often reduced beneficence to a proxy for therapeutic hope or functional enhancement. Other conceptions of beneficence—such as those involving broader social or relational considerations^63^—were notably absent. Of the 66 studies, only 9^8,28,31,33,39,45,49,59,61^ reported evaluating QoL before treatment, while 15 assessed it post-treatment^8,39–43,45,49,51,53,59,64–67^. Among these, 9 studies used standardized QoL scales (QOLIE-31, QOLIE-89) to compare pre- and post-treatment outcomes across studies^8,39–43,45,53,67^. Despite limited discussion of QoL in the literature, all studies employing QoL scales reported significant improvements (Table 2).

**Table 2:** Ethical recommendations for the development and implementation of closed-loop neurotechnologies

Theme	Recommendation	Key actions
Collaborative development	Establish inclusive and interdisciplinary working groups	Form international groups with diverse representation. Prioritize cross-disciplinary collaboration.
	Implement iterative and participatory development	Circulate drafts to key stakeholders (patients, industry, organizations). Refine guidelines based on real-world testing.
Evidence-based frameworks	Develop evidence-based and culturally adaptive guidelines	Involve AAN, AAS, PAHO to promote global applicability. Tailor frameworks to cultural and healthcare system differences.
	Integrate quantitative and qualitative evidence	Fund both qualitative and standardized quantitative research. Encourage journals to publish mixed-method studies.
Accountability and dynamism	Standardize transparent reporting and accountability	Develop ethical documentation templates using ICMJE and COPE protocols. Require adoption by FDA, EMA.
	Aim for dynamic and adaptive guidelines	Host regular forums for ongoing review and updates. Involve IEEE and major conferences.
Equity and sustainability	Address global equity and access	Reduce disparities in CL neurotechnology access. Engage advocacy groups and implement financial assistance models.
	Assess sustainability and environmental impact	Conduct lifecycle assessments in clinical trials. Collaborate with CCAC to minimize carbon footprint and align with climate goals.
Capacity building & public engagement	Strengthen education and training	Develop training programs for researchers, clinicians, and review boards. Provide workshops and toolkits via IBRO, IBI, INS.
	Foster public engagement and awareness	Host public forums (GESDA, WEF). Run campaigns to demystify CL neurotechnologies and amplify patient voices.

*CL* Closed-Loop, *INS* International Neuroethics Society, *SfN* Society for Neuroscience, *WHO* World Health Organization, *AAN* American Academy of Neurology, *AAS* African Academy of Sciences, *PAHO* Pan American Health Organization, *FDA* Food and Drug Administration, *EMA* European Medicines Agency, *IBRO* International Brain Research Organization, *IBI* International Brain Initiative, *CCAC* Climate and Clean Air Coalition, *GESDA* Geneva Science and Diplomacy Anticipator, *WEF* World Economic Forum, *ICMJE* International Committee of Medical Journal Editors, *COPE* Committee on Publication Ethics.

### Nonmaleficence

Nonmaleficence, the ethical obligation to avoid causing harm, requires that medical interventions do not inflict more harm than benefit^26^. Depending on the specific kind of harm—whether physical, psychological, or identity-related—different strategies may be necessary to address and mitigate risk. The reviewed literature reported and discussed a range of adverse effects and safety concerns associated with CL systems. Reported side effects varied from minor discomfort to severe complications, occasionally necessitating device removal. Fifty-six of 66 studies addressed adverse effects^32,40,48,50,66,68–72^. Among them, 15 studies referenced side effects of alternative treatments^9,27–30,32–34,43,46,57,73–76^, while 43 studies documented side effects specifically linked to CL neurotechnologies. Of the studies reporting CL-related adverse effects, 21 attributed them to the device or stimulation itself^9,28,31,35,37–39,41,42,49,53,55,62,64,65,73,75,77–80^, while 7 identified complications arising from implantation surgery^39,41,42,45,56,64,81^. System removal was reported in 8 studies^8,31,37,39,43–45,60^. Six studies intentionally induced transient side effects such as phosphenes, changes in color vision, paresthesia, epigastric discomfort, and hot flushes to establish patient-specific thresholds or confirm electrode positioning^9,35,49,78,82^. One study also explored using these side effects as a warning system for patients^65^.

Adverse events related to suicidality were reported in 4 papers^37,39,41,42^. Geller et al.^37^ specifically reported three cases of mental health deterioration during a clinical trial, with one participant with a history of depression and suicidal ideation dying by suicide and the other two reporting depressive episodes and suicidal thoughts potentially linked to the device. It should be noted, however, that both of them had prior histories of depression, with one having previously attempted suicide^37^. In contrast, Morrell et al.^42^ found no significant difference in suicidality-related adverse events between the treatment and sham groups. All six affected individuals had a history of depression or anxiety or met depression criteria on the BDI-II or CES-D. One participant died by suicide four weeks after stimulation began^42^. Nair et al.^41^ reported that 9.8% of participants experienced suicidality-related adverse events, including suicidal thoughts, behaviors, and attempts, with most having a history of depression. Two patients undergoing brain-responsive neurostimulation died by suicide, one of whom also had a history of suicidality^41^. Tager et al.^39^ discussed a similar case and explored possible explanations, including the hypothesis that as mood improves, patients may experience increased energy levels, which could heighten suicide risk in those with a history of depression and suicidality^39^. The authors called for further research on whether neuromodulation might contribute to suicidality in certain patients. Even anecdotal reports of RNS users with pre-existing suicidal thoughts experiencing worsening symptoms warrant close monitoring, even when mood symptoms appear resolved^39^.

Overall, avoidance of bodily harm and the safety of the CL systems represented a major concern in the reviewed literature, with 29 studies addressing various safety aspects. Although safety claims are widespread, the rigor of assessments varies. About one-third of studies asserted system safety^8,36,37,40,41,43,45,51,61,64,66–68,74,81^, but only five referenced safety assessments via regulatory bodies such as the FDA^41,43,51,66,74^. Eighteen studies conducted independent safety assessments^8,31,33,37,41–43,45,46,59,60,65,67,75,78,82–84^, while four discussed unspecified safety measures^53,67,78,82^. Three studies reported suboptimal safety^31,75,85^, and two called for further research to fully assess safety^75,85^. 12 studies discussed further concerns with potential forms of harm associated with CL systems^27,28,39,43,45,47,56,64,65,75,77,81^. These concerns ranged from discomfort and increased patient burden to stigmatization due to device visibility^56^. Cernera et al.^27^ described how the system can cause discomfort and restrict movement, while users must frequently reposition sensors, sometimes multiple times per day^27^. In the context of side effects, two studies also raised concerns about non-effectiveness in individual patients, highlighting that, even in the eyes of researchers, potential harm needs to be weighed against the technology’s benefits^28,39^. Swann et al.^28^ for instance noted that while deep brain stimulation (DBS) is effective for Parkinson’s disease, its efficacy varies. Programming requires a trained specialist, which can be time-consuming, and in some cases, optimal settings are never achieved^28^. Concerns about side effects and co-occurring disorders were also noted. Singh et al. highlighted a higher infection risk in pediatric patients, particularly those with autism or obsessive-compulsive disorder (OCD). While their study did not observe an increased infection rate, they recommended future research investigate this potential risk^81^.

Going beyond physical harm, the potential impact of CL systems on patients’ sense of identity remained largely unexplored. While the reviewed literature focused on therapeutic outcomes and life changes, no studies evaluated how these systems affect core aspects of a patient’s identity. Mental integrity, defined as the preservation of an individual’s cognitive and psychological state^86^, including cognitive function, emotional stability, and overall mental well-being^87^, was also not explicitly addressed as such in the reviewed literature. 17 studies examined memory integrity though^8,36–46,65,67,84,85,88^. These studies assessed whether CL treatment affected memory performance, particularly in cases of pre-existing cognitive decline. Among them, 12 studies conducted neuropsychological evaluations of patients’ memory^8,36–39,42–46,84,88^, reporting mixed results: some observed memory improvements, while others noted declines, sometimes fluctuating within the same study. Notably, QoL scales used in these studies, such as QOLIE-31 and QOLIE-89, include memory-related items^8,39–43,45,53,67^. However, discussions did not explicitly link memory function to potential unintended memory manipulation or its broader implications for QoL.

### Autonomy and privacy

Autonomy is the ethical principle that upholds an individual’s right to make informed, voluntary healthcare decisions based on their values and beliefs, provided they receive all necessary information^26^. Cognitive liberty and patient self-determination are considered core aspects of autonomy and patient-centered care, ensuring individuals can make informed choices about their own bodies and lives^89,90^. Self-determination refers to a patient’s right to accept or refuse medical interventions, reinforcing the need to respect personal values in decision-making. Cognitive liberty extends this to include control over one’s cognitive processes and states of awareness, free from external interference. Despite their ethical significance, none of the reviewed studies explicitly discussed these dimensions of personal autonomy. However, some studies addressed autonomy issues in the context of obtaining written informed consent from study participants. While most studies reported obtaining consent, only five papers also examined the decision-making process, including patients’ need for information and their ability to provide informed consent^39,43,49,56,84^. One study evaluated patients’ decision-making capacity through neuropsychological assessments, leading to the exclusion of some participants due to concerns about their ability to consent^84^. Another study emphasized the importance of providing patients with information and acknowledged their reasons for choosing CL treatment^43^. While one study briefly mentioned that patient or family preferences were considered^60^, others stated that treatment options were extensively discussed^39,49,56^. Some studies also highlighted the lengthy decision-making process, with one reporting that discussions about the device and alternative treatments lasted for 10 months before surgery^43^.

While autonomy and privacy are analytically distinct ethical values, they increasingly converge in the context of digital health and neurotechnology—particularly where control over neural data becomes central to self-determination. As Vold and Whittlestone (2019) observed, in the era of digital data, autonomy is deeply entangled with privacy and individual control over access and use of one’s personal data^91^. This conceptual overlap provides the rationale for introducing privacy-related concerns within the same thematic domain, while also respecting their normative differences. Two studies addressed anonymity and confidentiality, stating that no identifiable data were included in the research^57,58^. Some studies implicitly referenced mental privacy concerns, defined as the protection of cognitive processes, inner thoughts, and emotional states. Given that CL systems involve continuous neurophysiological monitoring and real-time adaptive feedback, they are believed to pose a greater risk to mental privacy than non-neural technologies^92^. This trade-off between preserving patient privacy and maximizing device functionality exemplifies a broader issue of value conflict, which cannot be resolved through technical means alone but requires ethical deliberation involving principles such as proportionality, least-infringement, and stakeholder engagement. Thirty-nine studies reported data management practices that implicitly suggest the moral objective of privacy preservation, either during primary data collection or retrospective analysis^9,10,27–31,33–35,39,43,44,49–52,54–56,59–61,64,66,67,69,72–75,78–82,85,93,94^. Various external systems were used to download, store, and process collected data. However, only six studies specifically addressed patient privacy. Some focused on data security measures^55,81^, or ensuring that data remained inaccessible to the public^43^, while others explored embedded data processing as a privacy-enhancing solution^34,74,82^.

Ferleger et al.^74^ identified privacy risks such as personal data exposure and third-party interference, noting that fully implanted neurostimulation systems — which process data internally and automate stimulation — may have lower privacy and security risks than wearable or distributed systems^74^. However, the study also highlighted limitations of fully implanted devices, including restricted data recording and lower processing power, which hinder interpretability and functionality in chronic treatments^74^. This suggests a prima facie conflict between privacy protection and device functionality. Similarly, He et al.^34^ described data vulnerability in CL and adaptive DBS systems^34^. While these systems optimize energy consumption, minimize side effects, and prolong clinical efficacy, they rely on external sensors such as surface electromyography (EMG), wearable accelerometers, or electrocorticography (ECoG) to detect movement-related symptoms. The use of external sensors poses challenges, including reduced patient compliance, increased power demands, and—most critically—data vulnerability if communication with the pulse generator is compromised or hijacked.

### Justice

The principle of justice concerns the fair distribution of benefits, risks, and costs, ensuring equitable access to healthcare resources without disproportionately burdening or privileging any individual or group^26^. In the reviewed literature, implicit references to justice can be primarily associated with economic aspects, particularly the costs of CL neurotechnologies. While seven articles addressed this issue, their discussions were largely descriptive, providing cost-related information without ethical analysis^10,56,66,71,76,83,85^ One study suggested that technological advancements could help lower costs^71^, while three others identified high costs as a barrier to implementation^10,56,83^. Schneider et al.^56^ further argued that while costs limit applicability, economic considerations should not dictate medical decisions, emphasizing the role of insurance companies in determining coverage and reimbursement^56^. Some studies weighed costs against benefits. For example, while the AspireSR generator offers technical improvements over the Demipulse, it reported a lack of clarity on whether the added costs are justified by the clinical benefits of the CBSD feature in the short term^56,85^. Additionally, one study attempted a cost-effectiveness analysis of traditional versus CL vagus nerve stimulation, comparing overall healthcare expenditures, including emergency room (ER) visits and hospital costs, but found no significant cost differences^66^. None of the analyzed studies addressed the fair distribution of resources, such as funding, healthcare professionals, or technology, nor did they examine whether CL systems were accessible to all patients, regardless of socioeconomic status or geographic location. Similarly, no studies explicitly considered bias prevention, applicability across diverse patient demographics, inclusion and diversity in study populations, or other broader ethical implications related to fairness. These omissions highlight that justice-related concerns—particularly regarding equity, inclusion, and sustainability—are not being addressed through ethical analysis, but largely treated as technical or logistical challenges. To meaningfully engage with such issues, a robust ethical framework must not only recognize distributive justice as a core concern, but also provide mechanisms to manage trade-offs and value conflicts between economic feasibility, equitable access, and long-term impact.

### Patients’ lived experience

Patients’ lived experience encompasses their subjective perceptions, emotions, and personal insights as they navigate healthcare systems and manage their conditions. Understanding these experiences is crucial for developing patient-centered technologies, as patients occupy a unique epistemic position with regard to their illness that demands recognition^95^). Moreover, lived experience may surface ethically relevant concerns that elude standard principlist categories—for example, relating to social vulnerability, the relational nature of care, or impacts on self-understanding. These dimensions often align more naturally with alternative ethical frameworks, such as the ethics of care, which emphasize interdependence, relationality, and context-sensitive responsiveness to patients’ needs. These factors explain the emergence of the lived experience as a fifth thematic family beyond the conventional principlist framework, to allow for the ethical significance of phenomenological and relational dimensions.

In the reviewed articles, the impact of neurological and psychiatric conditions on identity, social life, and daily functioning was discussed by over one-third of the studies. Many highlighted how these conditions affect social relationships, self-perception, and daily routines^8,27,28,30,32–34,36,39–43,45,49,55,58,60,61,65,68,77,80^. Loring et al.^40^ emphasized the profound impact of uncontrolled epilepsy on self-identity, noting that mood and social comorbidities significantly influence quality of life (QoL). They suggested that simply reducing seizures may not lead to sustained QoL improvements, particularly for long-term epilepsy patients with limited time for meaningful life changes^40^. Similarly, Opri et al.^33^ highlighted the severe effects of essential tremor (ET), linking it to progressive functional impairment, social stigma, and depression, with 65% of individuals with upper limb tremor experiencing serious daily challenges^33^. Conversely, O’Donnell et al.^43^ documented significant improvements following therapeutic interventions. One epilepsy patient, previously suffering from daily absence seizures, benefited from the ANT-RNS system, which enables remote monitoring via ECoG data, reducing unnecessary hospitalizations and emergency medevac flights. Another patient, who experienced 25 seizures per month, remained seizure-free for nine months post-implantation. As a result, her hospitalizations significantly decreased, and her antiseizure medications (ASMs) were gradually reduced in preparation for a future pregnancy^43^.

Mood-related questions are typically included in QoL assessments, yet only three studies reported no stimulation-induced mood effects^37,41,42^, while one noted a slight mood decline^39^. Among the reviewed studies, 41 included patient or family reports^8,9,28,29,31,33–43,45,47,49,51–55,58–61,64–68,74–76,78,81,83,84,96^. Of these, nine focused on patients’ and caregivers’ reports on seizure frequency and duration, offering insight into subjective experiences and seizure patterns^47,51,52,55,60,66,68,81,96^. These reports provide insight into the patients’ subjective experience, highlighting differences in seizure patterns and the impact on daily life. Additionally, two studies noted self-reported QoL improvements^43,64^. More than half of the studies examined patients’ reports of adverse events, treatment tolerability, and system tolerability^8,9,28,31,33–43,45,49,52–55,58–61,64,65,67,74–76,78,83,84^. Some reported that patients did not notice the system^28,36,64^, whereas others found that patients could distinguish when the system was on or off^49,74,75^. One study disclosed not retrieving any data regarding the “patients ´awareness of stimulation”^67^, highlighting a neglected phenomenological aspect in current research. System noticeability extends beyond sensory perception to its physical visibility, which can be apparent to both patients and others. Schneider et al.^56^ cautioned that scars and visible components may cause patient discomfort and stigmatization. They emphasized the need to address cosmetic concerns, particularly when treating children^56^. Another key aspect of patients’ lived experiences is their decision-making regarding system use. Herron et al.^49^ described a patient who chose not to activate the system due to concerns about frequent battery replacement surgeries. The study suggests that for some patients, battery conservation may be more important than tremor reduction, meaning CL systems do not necessarily need to match the efficacy of open-loop systems to be appealing^49^. Understanding these individual priorities is crucial for developing patient-centered treatments and improving adherence and satisfaction.

Finally, AI-specific ethical considerations consistently appeared across ethical domains. In particular, algorithmic transparency emerged as a cross-domain ethical issue, with implications for autonomy, fairness, and trust. In some cases, value conflicts emerged between protecting patient data and preserving proprietary algorithms, raising complex trade-offs that are not easily resolved within a single principlist lens but demand broader normative engagement.

**Fig. 1:**
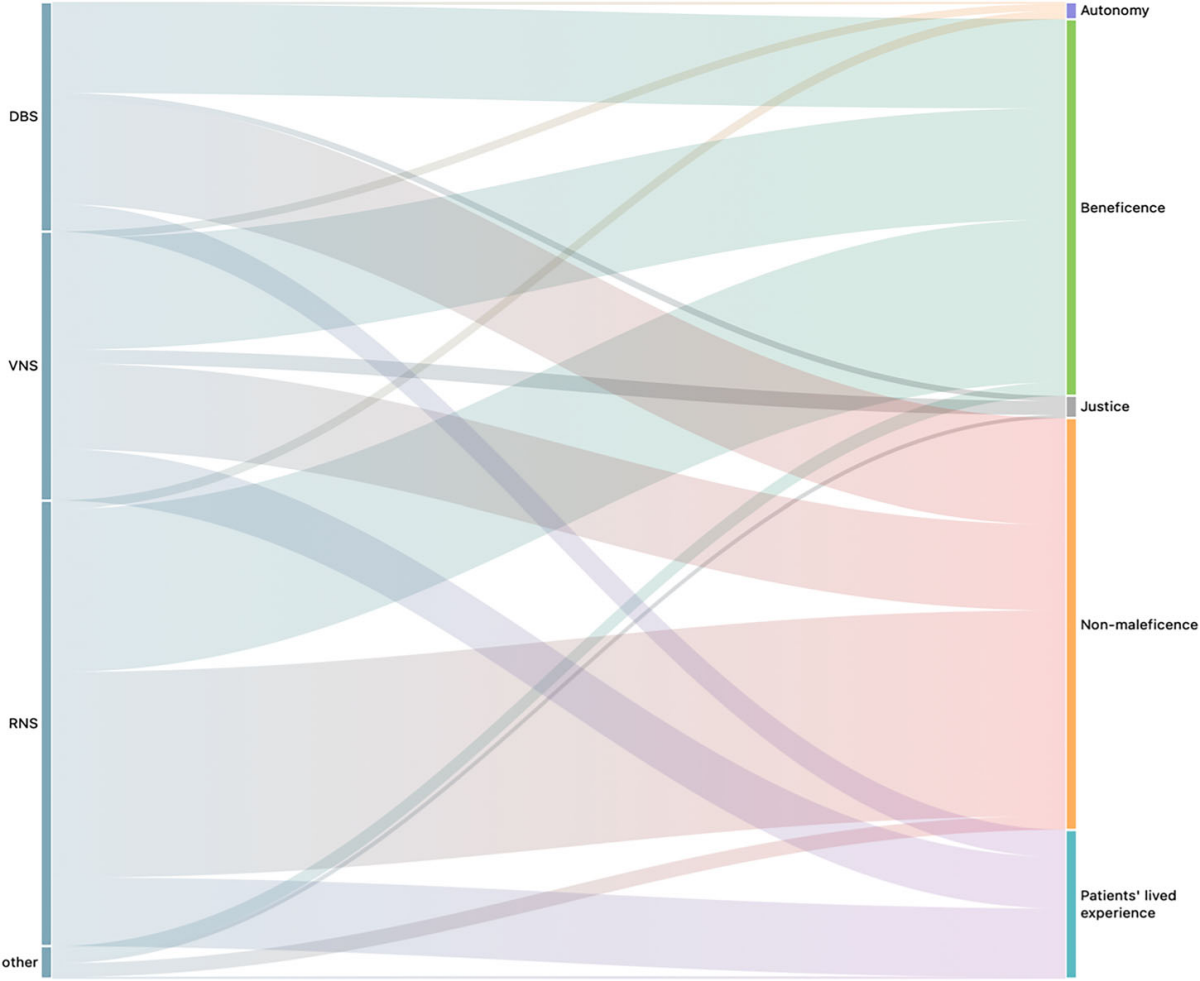
Alluvial diagram showing existing relationships between stimulation methods and ethical considerations. Bar thickness reflects the frequency of codes. ‘Other’ encompasses systems not categorized under responsive neurostimulation (RNS), deep brain stimulation (DBS), or vagus nerve stimulation (VNS).

Our findings indicate that explicit ethical assessments are rare in the existing literature. However, implicit considerations of beneficence and nonmaleficence appear more frequently, often reflected in reports of positive intervention outcomes or adverse effects in CL studies. In contrast, justice, privacy, autonomy, and patients’ lived experiences are seldom addressed. This shared ethical landscape was observed across three main families of CL neurotechnologies: responsive neurostimulation (RNS), deep brain stimulation (DBS), or vagus nerve stimulation (VNS), as outlined in Fig. 1.

## Discussion

CL neurotechnology systems are increasingly used to treat neurological and psychiatric disorders, yet ethical considerations remain largely unaddressed in clinical studies. Of the 66 analyzed studies, only one explicitly dedicated a section to ethics. Most studies approach ethics procedurally, focusing on ethics approvals and informed consent, rather than engaging in substantive ethical analysis. This reveals a recurring disconnect between regulatory compliance and deeper ethical reflection, which is needed when dealing with technologies that can directly affect mood, cognition, and identity. Beneficence and nonmaleficence appear often reduced to narrow clinical metrics or procedural benchmarks, such as treatment efficacy or adverse event frequency. Furthermore, they are addressed in an overly technical manner, with limited reference to substantive normative debates. Broader neuroethical issues such as mental integrity, mental privacy, security risks, and accessibility are rarely explored^86,97,98^. This raises questions about how such ethical considerations can be adequately incorporated into neurotechnology development and trial validation practices. The adaptive nature of CL systems, which continuously adjust based on real-time monitoring, introduces complex ethical challenges that extend beyond standard procedural ethics^99^. For instance, long-term effects on mental health, unintended behavioral changes, and the ethical implications of processing neural data should be more central to CL technology discussions^19,22,24^. Addressing these challenges may require integrating qualitative assessments to capture patients’ subjective experiences, alongside standardized quantitative measures. Additionally, reports of adverse psychological outcomes, including suicides among CL device users, suggest the need for enhanced monitoring and psychological support, particularly for patients with pre-existing psychiatric conditions^100^. Evaluating whether such individuals require additional care could help mitigate risks and improve CL system safety. These limitations in current reporting and ethical engagement form the empirical basis for the recommendations presented in the next section. Rather than being predetermined or conceptually abstracted, the recommendations emerge inductively from the patterns, omissions, and tensions identified in our thematic analysis.

Another critical research ethics challenge emerging from our analysis is the sporadic consideration of patients’ lived experiences and the phenomenology of using these advanced systems. Current clinical trials often focus predominantly on quantitative outcomes such as seizure reduction, motor improvement, or cognitive enhancement, while overlooking the subjective experiences of the patients undergoing these interventions. The lived experience of having one’s brain modulated by an implantable or wearable device, particularly in a CL system that continuously adapts without conscious input, can have profound implications for a patient’s sense of self, autonomy, and mental agency. Research ethics requires that trials consider not only the physiological outcomes of treatment but also the phenomenological dimensions—how patients perceive and interpret these technologies’ effects on their cognition, emotions, and identity. Without thorough engagement with these subjective experiences, researchers may miss critical insights into how patients adjust to these technologies, which could affect treatment compliance, satisfaction, and long-term mental health outcomes. Engaging with patients’ narratives and developing better quantitative-qualitative assessments to be integrated into clinical trials can provide a fuller picture of the ethical implications of CL systems and offer valuable feedback for improving the patient-centeredness of these interventions.

Given the implications CL technologies can have for mental integrity and personal autonomy^19^, it is crucial to transparently inform patients about the use of their neural data in CL neurotechnology applications to foster trust, transparency, and informed decision-making. Inadequate consideration of neuroprivacy poses a significant research ethics concern. Beyond simply ensuring that data is anonymized and stored securely, there is a need to scrutinize how neural data is shared, who has access to it, and how it might be used beyond the clinical context. For instance, there is a possibility that neural data could be commercialized or accessed by third parties, such as insurers or employers, in ways that undermine patient autonomy^92^. Research ethics requires a proactive approach to safeguarding privacy, which involves not only securing patient consent for data use but also implementing robust measures to promote that the data remains private and that patients retain control over it. Transparency around the use of AI algorithms is especially critical. Algorithmic processing plays a central role in how stimulation is adjusted in CL systems, yet many of the studies failed to describe how these algorithms work, how decisions are made, or how transparency is maintained. Patients and researchers alike should have access to information about how these algorithms function, what data they use, and how they might influence patient outcomes. Ethical challenges related to algorithmic opacity, explainability, and value alignment are rarely addressed in the literature. These issues not only intersect with autonomy and privacy but may also create normative tensions that require careful conflict resolution such as when data utility and transparency conflict with the protection of sensitive neural information. Without adequate inspection and accountability regarding these algorithms, we risk introducing biases or errors that could harm patients or compromise the efficacy of treatment.

Connected to algorithmic transparency is the risk of bias in the automated data processing and decision-making algorithms used in these systems. Machine learning algorithms for CL systems, often trained on limited or homogenous data, may not work equally well for all demographic groups, leading to unequal or harmful outcomes^101^. Bias in algorithm design or data selection can result in certain populations receiving less effective treatment^101^. Clinical trials must address this by design through best practices such as employing diverse datasets, ensuring fairness in algorithmic performance, and actively mitigating biases. If undetected, biases can reiterate or amplify discrimination risks, as CL systems may unintentionally reinforce healthcare disparities. Certain groups could be excluded from trials or receive suboptimal care if algorithms are not designed in a manner that inclusively considers their specific neural patterns. For example, individuals with atypical neural activity—whether due to neurological conditions, disabilities, or simply natural variations—might receive less effective treatment or be excluded from clinical trials altogether. This could exacerbate existing disparities in healthcare access and quality. To prevent discrimination, trials should prioritize inclusivity and fairness in both participant recruitment and algorithm testing. Transparent and adjustable automated systems are essential to ensuring that CL neurotechnologies are equitable and benefit all patient populations.

In addition to these individual-level concerns, broader societal and global ethical considerations appear unaddressed in the current ethical frameworks governing clinical trials of CL neurotechnologies. Access to advanced CL treatments is likely to be unequal, with disparities between high-income and low-income countries and within underserved populations in wealthier regions^98,102^. Addressing these disparities requires an ethical framework that considers issues of equity and inclusion, ensuring that the benefits of neurotechnology are not confined to privileged groups. Furthermore, financial barriers, particularly in healthcare systems that do not cover experimental treatments, must be factored into discussions about access and fairness.

Lastly, another significant but little-studied ethical issue is the effect that the development and application of closed-loop neurotechnologies will have on the environment^103^. CL systems have a significant carbon footprint because they frequently rely on machine learning and ongoing data processing. Adopting instruments to quantify and evaluate effects as well as incorporating sustainability concepts into the development and application of these technologies are necessary to address these environmental issues. To enable the responsible and successful application of these systems, neuroengineers, clinicians, researchers, ethicists, and other stakeholders will need to work together to develop a comprehensive ethical framework that takes equity, inclusion, sustainability, transparency, and patient-centeredness into account.

### Towards ethical recommendations for CL systems

As the use of CL neurotechnologies expands in the treatment of neurological and psychiatric disorders, the need for a coherent, globally accepted ethical framework becomes paramount. Ethical oversight promotes responsible use of CL technologies and safeguards the rights and well-being of participants. In particular, the integration of AI into CL systems introduces new layers of ethical complexity. Challenges such as algorithmic opacity, explainability, bias, and value alignment are not only technical issues but deeply normative ones. They cut across key ethical principles—impacting autonomy (through lack of algorithmic transparency and informed understanding), nonmaleficence (through potential algorithmic error and harm), and justice (through unequal model performance or structural bias). Importantly, our review found that these dimensions remain almost entirely unaddressed in clinical trial reporting and require direct attention in ethical guideline development.

The ethical issues identified in the preceding analysis—particularly those related to gaps in reporting, lack of patient-centered perspectives, and limited engagement with substantive ethical principles—point to structural weaknesses in how ethics is integrated into clinical research practice. These observations motivate the need for systemic responses that go beyond individual study design. The following recommendations are thus intended to operationalize ethically responsive practices at a broader institutional level, while remaining informed by the specific shortcomings and lived concerns identified in the literature. Rather than offering an abstract or pre-defined roadmap, the following recommendations emerged inductively from the gaps, themes, and blind spots identified in our review. Rather than constituting a fixed or exhaustive set, they are intended as actionable responses to the challenges identified through thematic analysis but are also mindful of normative insights from a broader range of ethical perspectives not exclusively reducible to the principlist framework. This meta-level strategy seeks to promote ethical guidelines that are not only effective and widely endorsed by researchers, clinicians, ethicists, policymakers, and patient representatives but also serve as a flexible practical framework to guide researchers and companies in the validation and assessment of their technologies. We outline ten actionable steps that the scientific community can undertake to develop ethical recommendations that reflect the diverse perspectives and needs of the neurotechnology community. These recommendations aim to guide researchers, clinicians, industry and other stakeholders, in the validation, assessment, and implementation of CL systems.

1. **Establish Inclusive and Interdisciplinary Working Groups:** The establishment of multidisciplinary working groups comprising neuroscientists, physicians, ethicists, patient advocates, legal professionals, and legislators must be the first step in creating community-agreed ethical standards. These groups should also include AI ethics experts to ensure that algorithmic transparency, explainability, and fairness are considered from the outset. Interdisciplinary working groups should be empowered to identify and negotiate ethical priorities through an open-ended, deliberative process. Rather than beginning with fixed principles, they should allow space for consensus to develop through inclusive discussion. To uphold representation across disciplines, geographical areas, and demographics, these working groups can be established by international organizations and professional associations like the World Health Organization (WHO), the Society for Neuroscience (SfN), the Federation of European Neuroscience Societies (FENS), the International Federation for Clinical Neurophysiology (IFCN), the International Brain Initiative (IBI) and the International Neuroethics Society (INS). International and interdisciplinary collaboration is essential to tackle the complex issues presented by CL systems^19^. First steps in this direction have already been made, for instance in the context of the NIH BRAIN Initiative^104^ and the International Brain Initiative^105^. The formation of interdisciplinary governance groups is intended as a flexible, context-sensitive process. These groups should be empowered to identify and negotiate ethical priorities based on empirical realities and stakeholder input. Rather than prescribing a fixed set of principles in advance, the process should remain open to diverse normative perspectives—including principlist, relational, procedural, and justice-based frameworks—and focus on reaching ethically justified consensus through inclusive deliberation.
2. **Create Iterative and Participatory Development Processes:** Ethical guidelines must be developed iteratively, incorporating stakeholder feedback at every stage. Drafts should be reviewed by patient advocacy groups, industry leaders, and global health organizations to advance broad representation. Pilot projects should test draft guidelines in real-world clinical trials, with funding support from funding agencies such as the NIH BRAIN Initiative, ERANET Neuron or Horizon Europe. Insights from these trials should be used to refine recommendations based on practical outcomes. Working groups can leverage existing frameworks for stakeholder involvement in healthcare guidelines^106^ to structure this process^105^. This process should explicitly integrate feedback on how AI components of CL systems affect trust, consent, and perceived agency. Furthermore, it should include procedures for identifying value conflicts—such as between privacy and functionality—and for facilitating transparent decision-making processes that aim for context-sensitive value alignment among stakeholders.
3. **Develop Evidence-Based and Context-Sensitive Frameworks:** Ethical frameworks should be grounded in empirical evidence, drawing from meta-analyses, original studies, and real-world data on the ethical challenges of CL systems. To encourage that cultural and healthcare system differences are considered, regional organizations—such as the African Academy of Sciences (AAS), PAHO, AAN, and the ASEAN Neurological Association—should guide the adaptation of these frameworks to local contexts. Tailoring ethical guidelines to specific cultural perspectives, including those of Indigenous communities, enhances their global applicability while respecting regional value-pluralism^105,107–109^. Ethical frameworks must also address regional variation in algorithmic fairness, privacy regulation, and digital literacy to ensure justice and inclusive AI governance. Cross-national organizations such as the International Brain Initiative can take the lead in harmonizing these guidelines internationally and balancing regional value-pluralism with cross-national agreement^105^.
4. **Promote Integration of Quantitative and Qualitative Evidence:** Guidelines should support clinical trials that integrate both standardized quantitative metrics (e.g., efficacy, safety) and qualitative insights (e.g., patient narratives, lived experiences). Qualitative insights can help illuminate patients’ understanding of AI-driven decisions, their trust in adaptive systems, and their concerns about algorithmic opacity. This integration should include co-production of research design, study questions, and protocols with participants and other stakeholders. Funding bodies can prioritize support for qualitative research (which remains underfunded), while neuroscience and neuroengineering journals can promote publication of studies using mixed methods. Including diverse forms of evidence helps align neurotechnology development with real-world patient needs and ethical considerations^110^. Furthermore, we recommend integrating patient-centered ethical perspectives into clinical development processes through continuous dialogue with stakeholders, including co-creation of research design, research questions, and study protocols to ensure responsiveness to local needs and patient experiences.
5. **Standardize Transparent Reporting and Accountability Mechanisms for AI Use:** Clear, standardized protocols for documenting ethical considerations in CL neurotechnology trials should be developed by organizations such as the International Committee of Medical Journal Editors (ICMJE) and the Committee on Publication Ethics (COPE), drawing from their existing instruments^111,112^. These protocols could mandate detailed reporting on informed consent processes, data privacy measures, risk management strategies, and impact on the patient’s subjective experience. Regulatory agencies like the FDA and the European Medical Agency (EMA) could incorporate these standards into clinical trial approval requirements, ensuring transparency and accountability. In addition, as many CL systems rely on AI-driven algorithms to process neural data and modulate stimulation, ethical guidelines should include specific provisions for algorithmic transparency and oversight. These include disclosure of model structure and training data, explainability limitations, fairness evaluations, and performance variability across demographic groups. The risks associated with algorithmic opacity, automation bias, and value misalignment must be ethically assessed—not just technically managed. Regulatory bodies should encourage or require algorithmic documentation (e.g., model cards), impact audits, and conflict-of-interest declarations. Particular attention should be given to ethical tensions between algorithmic optimization and core values like patient autonomy, privacy, and equity, which require normative deliberation beyond compliance checklists.
6. **Enable Dynamic and Adaptive Guidelines:** Given the rapid evolution of neurotechnology, ethical guidelines must be continuously updated to address emerging challenges. Conferences such as the International Brain Stimulation Conference, NeurIPS, SfN, and the Annual INS Meeting should host dedicated forums for reviewing and revising guidelines. Standardization bodies like the IEEE Standards Association can establish formal mechanisms for periodic updates, ensuring that ethical recommendations remain relevant, adaptive, and enforceable. Ongoing updates should reflect evolving standards for algorithmic accountability and AI ethics in neurotechnology.
7. **Address Global Equity and Access**: Access to CL neurotechnologies is likely to vary significantly between high-income and low-income regions^113,114^. Advocacy groups like Global Access in Action and global organizations like the United Nations Development Program could lead initiatives to reduce access disparities. Ethical frameworks should include provisions for equitable benefit distribution, such as financial assistance models, technology sharing, and open licensing of key components to expand accessibility for underserved populations. To address equity, sustainability, and potential value conflicts, ethical governance frameworks should combine principle-based reasoning with deliberative and participatory methods. This includes not only defining ethical priorities but also creating transparent processes for resolving conflicts—such as tensions between distributive justice and innovation incentives, or between environmental impact and therapeutic potential. Attention must also be paid to how algorithmic systems may replicate or amplify structural inequalities across different populations.
8. **Include Sustainability and Environmental Impact Assessments:** The environmental impact of CL systems, especially those reliant on deep learning, large-language models (LLMs) and continuous high-volume data processing, must be addressed in ethical guidelines^115^. Adherence to the UN Sustainable Development Goals could become routinely integrated in clinical trials involving CL neurotechnologies. Clinical trials could further incorporate lifecycle assessments of devices and algorithms to minimize carbon footprints, aligning with global climate justice goals. Organizations like the Climate and Clean Air Coalition (CCAC) could help establish sustainability benchmarks similarly to those developed for other technologies, while financial incentives could be developed to promote power-efficient solutions based on neuromorphic computing or other highly efficient brain-inspired architectures.
9. **Strengthen Education, Training, and Capacity Building:** To implement ethical guidelines effectively, the development of community-agreed training programs for researchers, clinicians, and institutional review boards is critical. Training should cover core AI ethics topics including algorithmic fairness, interpretability, and stakeholder accountability. Societies like the International Brain Research Organization (IBRO), the International Brain Initiative (IBI) and the INS can organize workshops and create open-access toolkits. These materials should cover key ethical principles, regulatory requirements, and practical strategies for applying the guidelines in clinical settings.
10. **Foster Public Engagement and Awareness:** It is critical that ethical guidelines reflect societal values, hence are rooted in meaningful public engagement. Hosting public forums through initiatives like the GESDA Annual Summit, the World Economic Forum (WEF) and patient organization such as the European Federation of Neurological Associations (EFNA) can foster dialogue between experts, patients and the broader community. Civil society organizations such as Patient-Centered Outcomes Research Institute (PCORI) can promote that patient voices are central to the development process^116^. Engagement activities should also explore public perceptions of AI in neurotechnology and identify concerns about data use, agency, and decision-making transparency.

## Methods

A comprehensive literature search was conducted on November 14, 2023 for English-language articles across the following four databases: *PubMed*, *Scopus*, *IEEExplore* and *PsycNet*. The search targeted titles and abstracts using the following query: “((Machine learning*) OR (artificial neural networks) OR (artificial intelligence) OR (deep learning) OR (pattern recognition) OR (predictive modeling)) AND ((closed-loop*) OR (adaptive brain stimulation) OR (brain-computer interface) OR (neural interface) OR (neurostimulation) OR (brain stimulation)OR (brain implant) OR (implantable neurostimulator)) AND ((depression) OR (schizophrenia) OR (Tourette’s) OR (epilepsy) OR (Parkinson’s disease) OR (essential tremor) OR (obsessive-compulsive-disorder) OR (neurology) OR (neuropsychiatry) OR (neurological disorders) OR (neuropsychiatric disorders) OR (psychiatry)) AND ((human subject) OR (clinical trial) OR (clinical study))”. In line with our research objective – to examine whether and how ethical considerations are addressed in reports of current clinical research on CL neurotechnology –, we deliberately excluded ethics-related terms (e.g., ‘ethic*’) from the search strategy. This was done to avoid restricting the dataset to studies with explicit ethical framing and to enable an unbiased assessment of the extent to which ethical issues are reported or overlooked in the clinical literature. The query logic was adjusted to align with the search criteria of each database. Additionally, search results were cross-checked against a recent review of CL brain stimulation devices^7^ to ensure comprehensive coverage. The study protocol was not preregistered.

**Fig. 2:**
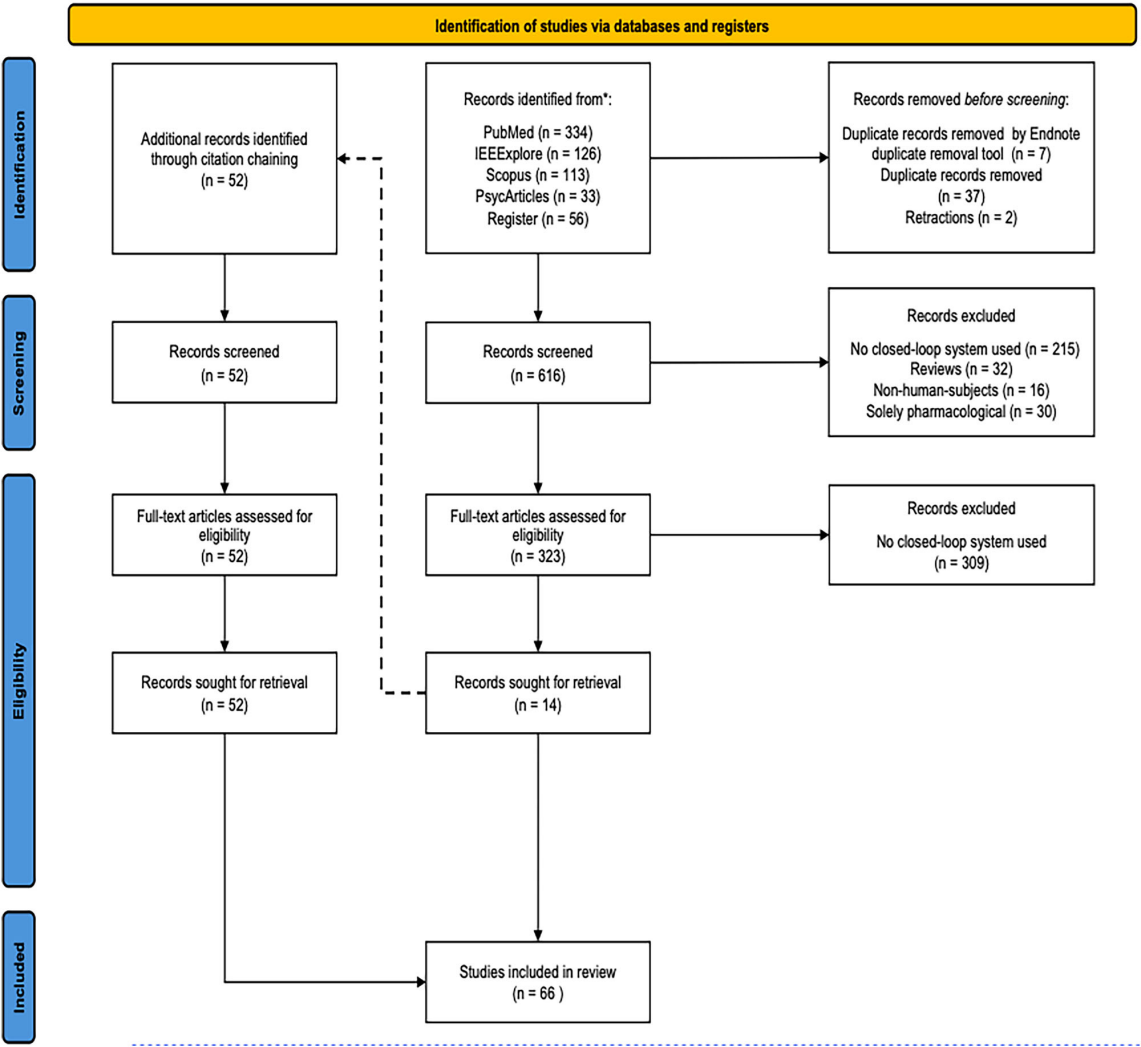
PRISMA Scoping literature review flow diagram. The diagram outlines the selection process of studies included in the review, detailing the number of records identified, screened, assessed for eligibility, and ultimately included, along with the reasons for exclusion at each stage.

A total of 662 papers were initially identified. All references were imported into EndNote for citation management. Two retracted papers and 44 duplicates were removed using EndNote’s Duplicate Removal Tool and manual screening. Eligibility was further assessed by reviewing the titles, abstracts, and full texts when necessary. Studies were included if they met the following criteria: (a) employed a closed-loop system; (b) involved human participants; (c) focused on neurological or psychiatric diseases; (d) were published between January 1, 2000, and November 15, 2023; and (e) were written in English. Given the heterogeneous terminology in the literature, CL systems were defined as necessarily incorporating: (i) real-time monitoring, (ii) immediate data processing, and (iii) adaptive feedback. Exclusion criteria included reviews, non-peer-reviewed articles, opinion letters, and studies using simulated data or non-human subjects (e.g., primates or rodents). To enhance comprehensiveness, citation chaining was performed in both forward and backward directions, identifying additional references that underwent the same screening and eligibility assessment. Ultimately, 66 papers were included in the final review. The search and eligibility assessment were performed by the first author and verified by the first author. Any disagreements were resolved by unanimous consensus among all authors (Fig. 2).

For analysis, we compiled a descriptive summary of the included studies, adhering to methodological recommendations for scoping reviews^117^ and categorized articles based on the type of CL system. We then performed a thematic analysis of the full texts, manually coding sections that addressed both explicit and implicit ethical considerations and grouping findings into broader thematic families. By explicit ethical considerations, we refer to instances where studies directly identify, discuss, and frame certain topics as ethical issues, explicitly recognizing them within an ethical framework. In contrast, implicit ethical considerations arise when studies address issues that have clear ethical implications—such as patient autonomy, privacy, or consent—without explicitly identifying them as ethical concerns. These cases often involve discussions of technical, clinical, or regulatory aspects that inherently carry ethical significance but are not framed as such. To uphold clarity and consistency in categorization, we applied predefined criteria to distinguish between explicit and implicit ethical discussions. Our overarching categories are based on the widely used principles of biomedical ethics by Beauchamp and Childress^26^, complemented by a fifth thematic family focusing on patients’ lived experience. We selected the principlist framework of Beauchamp and Chidlress as the starting point for our ethical analysis due to its widespread use and pedagogical prominence in clinical and medical ethics. As it is familiar to many researchers—particularly in Western biomedical contexts—it offers a shared reference point that facilitates systematic comparison across studies. Thematic analysis was carried out by [blinded] and checked by [blinded] using the qualitative data analysis software Atlas.ti for Mac version 24.1.1. Throughout our work, we adhered to the Preferred Reporting Items for Systematic Reviews and Meta-Analyses (PRISMA)^118^ and its extension for scoping reviews^119^ to ensure a systematic, transparent, and reproducible research process. These guidelines structured each stage of our review, enhancing methodological rigor and comprehensiveness. A flow diagram illustrating the full process is provided in Fig. 2. In a second analytical phase, we synthesized the coded material across categories and studies to identify recurring patterns, blind spots, and areas of ethical ambiguity or neglect. This inductive synthesis formed the basis for the recommendations presented in this paper. Rather than predetermined or abstracted in advance, these recommendations emerged directly from empirical gaps surfaced through our thematic coding.

We acknowledge several limitations. First, the diversity of terminology in CL neurotechnology may have resulted in the omission of relevant studies. To minimize this risk, we used a broad keyword strategy and triangulated results with citation chaining and recent reviews. Second, by restricting to English-language publications, we may have excluded valuable insights from non-English sources.

Third, our focus on human studies meant that preclinical and conceptual research was excluded—though we reference relevant neuroethical literature to fill this gap. Fourth, as with all scoping reviews, interpretive bias is possible; we mitigated this through double-screening, predefined criteria, and team cross-checks. Fifth, while principlism offers a structured analytic lens, it has been criticized for conceptual ambiguity^120^, limited practical utility^121^, and strong orientation toward Western, individualist moral reasoning^122^. In response to these concerns, recent scholarship has advocated for more inclusive approaches that integrate relational, cross-cultural, and patient-centered perspectives^123^. Our selection of the principlist framework was motivated by its pedagogical familiarity and sustained use in biomedical education and reporting. However, we recognize that alternative ethical paradigms—such as care ethics, relational approaches, and cross-cultural perspectives—may capture different dimensions of ethical relevance, particularly in the context of neural interface. To partially address this limitation, we included a dedicated results section on patients’ lived experiences. This dimension was added not only as a thematic family, but also as a normative response to the limitations of principlism. Lived experience can reveal ethical concerns related to self-identity, agency and stigma that are not easily subsumed under the four principles. Finally, given rapid developments in CL neurotechnology, the field is evolving quickly. We incorporated the most recent studies available at the time of review and highlighted future directions.

## Supplementary information

PRISMA-ScR-Fillable-Checklist-3

## Data Availability

All data generated and analyzed during this review are publicly available. The full raw dataset, including all coded references and thematic groupings, has been uploaded as a supplementary file and is also accessible via Zenodo at [10.5281/zenodo.15797207]. This dataset provides the necessary information to interpret, replicate, and build upon the findings reported in this article.
